# From adjustment disorder to schizotypal personality disorder

**DOI:** 10.1192/j.eurpsy.2024.1354

**Published:** 2024-08-27

**Authors:** C. De Andrés-Lobo, M. D. C. Vallecillo Adame, T. Jiménez Aparicio, A. Rodríguez Campos, N. Navarro Barriga, M. J. Mateos Sexmero, B. Rodríguez Rodríguez, M. Fernández Lozano, M. Calvo Valcárcel, M. Andreo Vidal, P. Martínez Gimeno, M. P. Pando Fernández, L. Rojas Vázquez, G. Lorenzo Chapatte, M. Ríos Vaquero, A. Monllor Lazarraga

**Affiliations:** ^1^Psychiatry, HCUV, Valladolid, Spain

## Abstract

**Introduction:**

Individuals with schizotypal personality disorder are characterized by tendencies to magical thinking, unusual perceptions, discomfort in social situations, and restricted affect. It is frecuent that they have social anxiety and have difficulty in understanding the motivations and thoughts of others.

**Objectives:**

Presentation of a case of a patient who was first diagnosed with adjustment disorder, but on a closer study, was discovered to have a schizotypal personality disorder.

**Methods:**

We conducted a bibliographic review by searching for articles about schizotypal personality disorder and theory of mind in Pubmed.

**Results:**

We present the case of a 39-year-old woman, diagnosed with adjustment disorder after a conflict at work with a colleague that caused her anxiety-depressive symptoms. In consultations, the patient shows verbiage without expansiveness or euphoria, with rambling speech. She expresses feelings of indignation and injustice, she is irritable, with contained anger. She refers that she prefers to be distrustful of others because she does not understand their intentions. Her thoughts are very rigid, which leads her to have avoidant and phobic attitudes, having no relationships of friendship throughout her life.

A neuropsychological evaluation is carried out, resulting in a surprising WAIS with a TIC of 128. However, the Mayer‐Salovey‐Caruso Emotional Intelligence Test (MSCEIT) shows difficulties in Perception, Comprehension and Emotional Management

Considering the patient’s symptomatology as a whole, it is noteworthy:

- Sustained social isolation throughout their life history
- Superficiality of interpersonal relationships
- Distrust and slight self-referentiality. Deficit in inferring the feelings and thoughts of others
- Peculiar speech with ideas of magical content, superstitions and rituals…

Which together supported a diagnosis of schizotypal personality disorder and generalized anxiety disorder. From this point we started to work on her self-esteem, modification of irrational beliefs and cognitive distortions, interpersonal communication and metacognitive therapy, with good results.

**Conclusions:**

The type of schizotypal patients who come to consultations most frequently are the actively isolated/timorous profile due to their intense social anxiety and difficulties in understanding and adapting to the social world around them. Initial therapy should be empathic support. The theory of mind is the ability to infer the other’s mental states and therefore predict their behavior, this ability being diminished in the schizotypal patient. Mentalization tasks, metacognitive therapy, cognitive flexibility training, social skills training, and promoting self-worth are useful. On some occasions it may be necessary to start psychopharmacological treatment to control anxiety and unusual perceptions when they cause discomfort.

**Disclosure of Interest:**

None Declared

